# Pelvic Floor Muscle Training Using the Perifit Device for the Treatment of Urinary Incontinence: A Pragmatic Trial Using Real-World Data

**DOI:** 10.1089/whr.2023.0172

**Published:** 2024-03-13

**Authors:** Erica T. Perrier, Louise Aumont

**Affiliations:** Department of Research & Development, X6 Innovations, Paris, France.

**Keywords:** biofeedback, mobile applications, pelvic floor, rehabilitation, urinary incontinence, women

## Abstract

**Introduction:**

There is a need for home-based alternatives for women to self-manage urinary incontinence (UI). Using a real-world data approach, the aim of this analysis was to evaluate whether training with the Perifit device was effective in reducing UI symptoms.

**Materials and Methods:**

A total of 6060 women (45 ± 10 years) with UI who purchased the Perifit device, completed a validated symptoms questionnaire before training (T1) and again at one or several predefined timepoints during training: T2, after completing 40–60 games; T3, after 90–120 games; and/or T4, after 280–300 games.

**Results:**

UI symptom score decreased progressively from 8.4 ± 4.8 points at T1; to 6.3 ± 4.7 points, 5.5 ± 4.5 points, and 4.6 ± 4.5 points at T2, T3, and T4, respectively (all *p* < 0.001). The percentage of respondents reporting objective improvement in UI symptoms increased from 71%, to 79%, to 85% at T2, T3, and T4, respectively. Effect size was medium (T2) to large (T3, T4). Higher symptom score at baseline was associated with higher likelihood of improvement. There was no effect of other characteristics including respondent age, menopausal status, time since childbirth, prolapse, or baseline strength on symptom improvement.

**Conclusions:**

This analysis of responses from over 6000 real-world users suggests that home training with the Perifit may be an effective way to reduce UI symptoms in women of all ages. Given the quality of life, economic, and social burdens of living with UI symptoms, home-based pelvic floor muscle training with the Perifit may be a promising tool to allow women to self-manage UI.

## Introduction

Pelvic floor muscle training (PFMT) is a form of exercise intended to strengthen and improve the function of the pelvic floor muscles. The pelvic floor muscles are a group of muscles located in the pelvis that support the pelvic organs, including the bladder, uterus (if present), and rectum. These muscles play a vital role in maintaining urinary and bowel continence, allowing sexual function, and providing support to the pelvic organs. The pelvic floor muscles can become weakened or function may be compromised due to various factors such as pregnancy, childbirth, age, obesity, chronic cough or constipation, physical activity, and certain medical conditions.^1^ Weak pelvic floor muscles are associated with issues such as urinary and fecal incontinence, pelvic organ prolapse, sexual dysfunction, and other issues.^2^

It is estimated that between 23% and 53% of women suffer from some form of urinary incontinence (UI),^3–6^ with wide-ranging impacts including poor quality of life,^5^ a modification or reduction in their physical activity,^7,8^ financial burden,^9,10^ and sexual dysfunction.^11,12^ PFMT is an effective and widely recommended conservative treatment for reducing stress urinary incontinence (SUI), urge urinary incontinence (UUI), and mixed urinary incontinence (MUI) in women, with systematic reviews and expert committees concluding that PFMT can significantly improve symptoms of UI and even eliminate or greatly reduce urine leakage in many women.^13,14^ The training helps to strengthen and improve coordination of the pelvic floor muscles, increasing their ability to support the bladder and other pelvic organs and prevent leakage during activities that may increase intrapelvic pressure.

PFMT, also known as Kegel exercises, involves the repeated contraction and relaxation of the pelvic floor muscles, including both short, intense contractions and longer, endurance-focused contractions. A significant advantage is that PFMT can be performed in nearly any setting, from structured visits with a pelvic health practitioner to home-based training where women simply contract and relax their pelvic floor muscles several times per day.

However, research has shown that not all women are able to perform an effective pelvic floor muscle contraction: a study providing verbal instruction on how to perform a Kegel found that only 49% of women were able to produce an “ideal” Kegel after being instructed on how to do so, while about 25% performed a movement that could potentially promote incontinence.^15^ Similarly, a second study found that 25% of women were unable to produce an elevating pelvic floor muscle contraction.^16^ This is especially relevant for home-based training, since roughly 2 out of 3 women with UI symptoms do not speak to a health care practitioner about their leak issues.^4,17,18^ There is therefore a real need to provide additional options to women who want to self-manage UI symptoms by performing proper PFMT.

Recently, at-home options including smartphone app-based guided exercise and force-sensing biofeedback devices have improved access to structured PFMT while also providing guidance and training programs to women who prefer to train in the privacy of their home. Use of these systems has the potential to improve access to and compliance with PFMT programs, and both app-based and app-and-probe-based systems have shown to improve UI symptoms in monitored conditions.^19–24^ Less is known about whether home-use of app-and-probe systems is effective at reducing UI symptoms in real-life conditions, in which an individual makes the decision to purchase an app-and-probe system for home use without clinical follow-up, or explicit or implicit reminders to train due to study participation.

One device available for home-use is the Perifit (www.perifit.co), which is FDA cleared to treat stress, mild-moderate urge, and mixed urinary incontinence in women, by strengthening of the pelvic floor muscles through exercise. The device provides biofeedback *via* smart phone technology. A key feature of the Perifit is that the user interface offers users a gamified experience, in which pelvic floor training is proposed in the form of short video games during which users control the game by contracting and relaxing the pelvic floor muscles to match programmed contract–relax patterns. It is available to be purchased over-the-counter and its gamified format may present an attractive and fun way to encourage regular pelvic floor exercise at home.

Furthermore, previous research has shown that the Perifit's force sensors can differentiate between a proper PFM contraction versus a straining or pushing movement,^25^ suggesting that it may be particularly helpful in encouraging proper PFMT in the home setting. However, to date, nothing has been published on its effectiveness in reducing UI symptoms. Using a real-world data approach, the aim of this analysis was to evaluate whether the Perifit device was effective in reducing UI symptoms in individuals using the Perifit probe for PFMT at home.

## Materials and Methods

This was a pragmatic analysis of real-world data generated by users of the commercially available Perifit device and its accompanying smartphone app. At no time were users of the device contacted by the research team. Before any analyses were performed, data generated by users of the commercially available device were de-identified of all personal information using the “Safe Harbor” method, in which a defined set of variables that could potentially identify an individual are removed.^26^ In accordance with 45CFR46.104(d)(4)(ii), this analysis was exempt of ethics committee approval as the identity of the device users could not be ascertained directly or indirectly through identifiers, the research team did not contact the device users, and the research team did not perform any activities that would allow for the re-identification of device users.

The Perifit smartphone app integrates an optional symptoms questionnaire that users can fill out to track their UI symptoms over time. The app is configured to propose the questionnaire to users at set times in their training program: at the very beginning of training, and at regular intervals afterward when users reach certain milestones of games played. Responding to this questionnaire is not mandatory; users are free to access the questionnaire at any time or to never access the questionnaire at all.

**FIG. 1.**
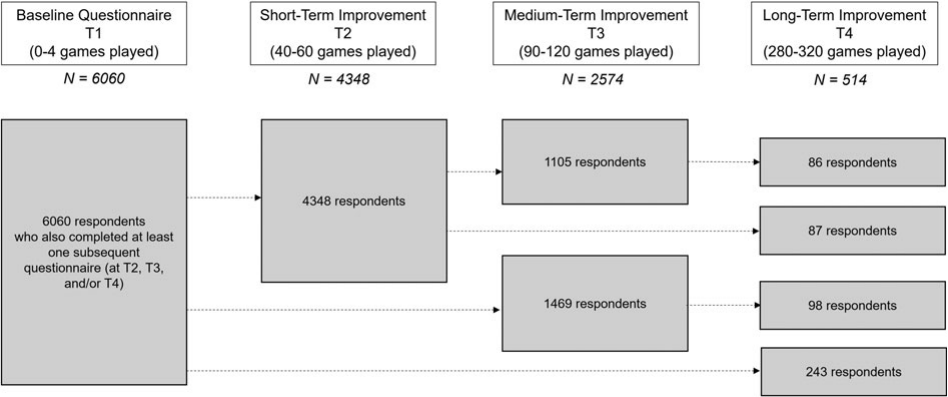
Schematic of questionnaire timepoints and number of respondents at each timepoint.

To generate the de-identified dataset, a custom code (Python 3.11, available at www.python.org) was written to extract data for individuals who met the following criteria: (A) women who started using the Perifit device between April 22, 2022 and May 24, 2023, and (B) who completed the in-app symptoms questionnaire before beginning to train with the device (Timepoint 1, or T1) and reported having symptoms of UI at the onset of their training, and (C) who also completed the questionnaire at one or more pre-defined time-points after training with the device (Timepoint 2, or T2, after completing 40–60 games; Timepoint 3, or T3, after completing 90–120 games; and Timepoint 4, or T4, after completing 280–300 games). This allowed us to assess short-term (T2), medium-term (T3), and longer-term (T4) outcomes related to the reduction in UI symptoms after training with the Perifit device (Fig. 1). To retain the most representative dataset possible of real-world users of the device, no eligible data were excluded based on other factors, such as gravity of UI symptoms, age, menopausal status, parity, or type of UI (SUI, UUI, or MUI)

UI symptoms were measured using the SUI and UUI/Overactive Bladder subscales from the Urinary Symptom Profile (USP), a validated assessment tool for urinary disorders and their severity.^27^ The Low Stream subscale was not included because it was not relevant to assess UI symptoms. Total score from the SUI and UUI subsections was computed to obtain a UI symptom score, with a higher score indicating greater symptom severity. A reduction in symptoms was defined as a decrease in UI symptoms as measured by a numeric decrease in UI symptom score derived from the questionnaire.

### Statistical analysis

All statistical analyses were performed in JASP (version 0.17.3.0, Amsterdam, NL, https://jasp-stats.org/). The change in UI symptom score between timepoints was assessed using paired-samples *t*-tests, on the condition that the normality assumption was met. Effect size for improvement in UI symptoms was estimated using Cohen's d. The percent of users reporting improvements (*i.e*., a numeric reduction in UI symptom score) is also reported for each timepoint. Subgroup analysis was also performed to assess potential baseline differences between users reporting improvement and those reporting no improvement in symptoms.

## Results

The final dataset included de-identified data originating from 6060 unique users of the Perifit device who reported UI symptoms at the start of their training (T1). Because completing the questionnaire is an optional part of using the device smartphone app, and because data were collected in a rolling manner, including users who began using the Perifit as recently as May 2023, and who therefore had reasonably completed less games, the number of questionnaire responses at each pair of timepoints varied. The final dataset included responses from 4348 users at T2, data from 2574 users at T3, and data from 514 users at T4.

**FIG. 2.**
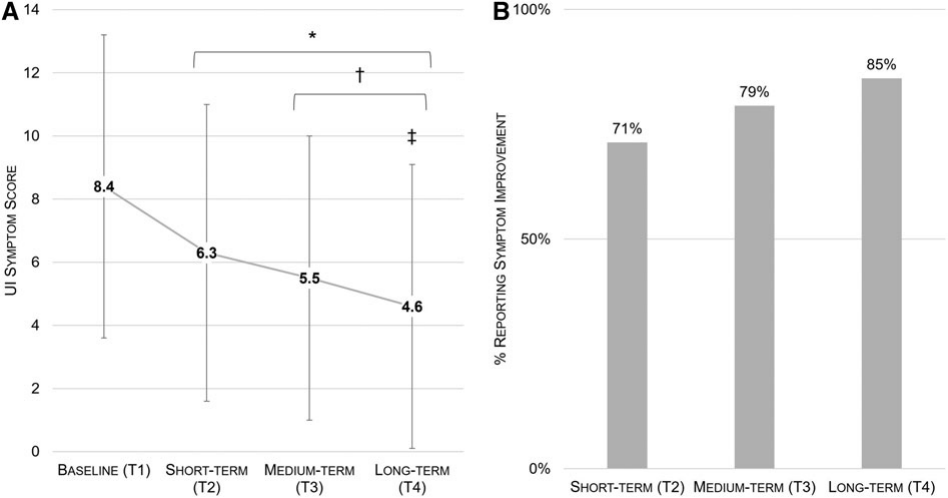
Evolution of UI symptom score **(A)** and % of respondents reporting symptom improvement over time **(B)**. *denotes significantly different from T1; ^†^denotes significantly different from T1 and T2; ^‡^denotes significantly different from all other timepoints. UI, urinary incontinence.

For all timepoints, UI Symptom Score and % respondents reporting improvement are shown in Figure 2. Results are reported by timepoint, below.

### Participant characteristics at baseline

Mean age of respondents was 45 ± 10 years (range, 18–87 years). Most respondents were premenopausal (61%), with 17% reported being postmenopause and 22% not declaring their menopausal status. At baseline, 4388 (72%) of users reported symptoms in both the SUI and UUI domains of the USP (known as mixed UI, or MUI). Symptoms of SUI only were reported by 832 users (14%), and symptoms of UUI only were reported by 840 users (14%). Mean UI symptom score was 8.4 ± 4.8 points at baseline.

### T2 results: short-term improvement

Overall, 3069 of 4348 respondents (71%) reported an improvement in UI symptoms at T2. Compared to baseline, mean UI symptom score decreased to 6.3 ± 4.7 points (*p* < 0.001 vs. T1). The effect size for symptom reduction was medium (Cohen's *d* = 0.669). On average, users responded after having completed 51 ± 5 games, with 41 ± 49 days elapsed since T1.

### T3 results: medium-term improvement

Overall, 2034 of 2574 respondents (79%) reported an improvement in UI symptoms at T3. Mean UI symptom score decreased to 5.5 ± 4.5 points (*p* < 0.001 vs. T1 and T2). The effect size for symptom reduction was large (Cohen's *d* = 0.881). On average, users responded after having completed 105 ± 8 games, with 62 ± 53 days elapsed since T1.

### T4 results: long-term improvement

Overall, 437 of 514 respondents (85%) reported an improvement in UI symptoms at T4. Mean UI symptom score decreased to 4.6 ± 4.5 points (*p* < 0.001 vs. all previous timepoints). The effect size was large (Cohen's *d* = 1.030). On average, users responded after having completed 301 ± 11 games, with 125 ± 70 days elapsed since T1.

### Subgroup analysis: responders versus nonresponders

To assess whether baseline differences may explain responders versus nonresponders, we conservatively categorized each subject as a “responder” only if their UI symptom score at their final response timepoint was less than their baseline score; and as a “nonresponder” if their UI symptom score at their final response timepoint was not less than their baseline score, regardless of what they had reported at any intermediate timepoints. Concretely, this meant that a user whose symptom score at T2 was less than their baseline, but whose symptom score at T3 or T4 had returned to baseline values would be counted as a “nonresponder” in the subgroup analysis.

We found that responders had higher mean symptom scores on both the stress and urge subscales of the USP (*p* < 0.001, Table 1), but were not different at baseline in terms of other continuous characteristics (age, baseline pelvic floor strength). We also evaluated possible differences in categorical variables (Table 2). We found no difference between responders and nonresponders in menopausal status, time since most recent childbirth, or presence of prolapse; all *p* > 0.05.

**Table 1.** Baseline Characteristics in Responders Versus Nonresponders, Continuous Variables

	*N*	Mean	Standard deviation	*p*-(difference between groups)
Baseline symptom score, SUI subscale				
Responders	4539	3.6	2.5	<0.001
Nonresponders	1521	2.9	2.4	
Baseline symptom score, UUI subscale				
Responders	4539	5.3	3.5	<0.001
Nonresponders	1521	4.0	3.5	
Year of birth (401 missing values)				
Responders	4254	1978	10	0.274
Nonresponders	1405	1978	10	
Baseline pelvic floor strength, grams				
Responders	4539	186	364	0.186
Nonresponders	1521	198	314	

SUI, stress urinary incontinence; UUI, urge urinary incontinence.

**Table 2.** Baseline Characteristics in Responders Versus Nonresponders, Categorical Variables

	Subgroup	Count (percentage)	*p*-(Chi square)
Menopausal status (missing 401 responses)			
Premenopausal (*n* = 3682)	Responders nonresponders	2771 (75%) 911 (25%)	0.471
Postmenopausal (*n* = 1010)	Responders nonresponders	746 (75%) 264 (25%)	
Unsure (*n* = 967)	Responders nonresponders	737 (76%) 230 (24%)	
Time since most recent childbirth			
Never (*n* = 847)	Responders nonresponders	620 (73%) 227 (27%)	0.441
In the past 8 weeks (*n* = 86)	Responders nonresponders	67 (78%) 19 (22%)	
More than 8 weeks but less than 1 year ago (*n* = 645)	Responders nonresponders	494 (77%) 151 (23%)	
One year ago or longer (*n* = 4472)	Responders nonresponders	3351 (75%) 1121 (25%)	
Prolapse			
No diagnosed prolapse (*n* = 5147)	Responders nonresponders	3871 (75%) 1276 (25%)	0.069
Grade 1 prolapse (*n* = 343)	Responders nonresponders	238 (69%) 105 (31%)	
Grade 2 prolapse (*n* = 414)	Responders nonresponders	312 (75%) 102 (25%)	
Grade 3 prolapse (*n* = 145)	Responders nonresponders	112 (77%) 33 (23%)	
Grade 4 prolapse (*n* = 11)	Responders nonresponders	6 (55%) 5 (45%)	

## Discussion

The main finding of this analysis was that women who performed structured PFMT exercises at home using the Perifit device experienced a reduction in UI symptoms, with significant improvements observable within 6 weeks of beginning to train. The percentage of respondents who reported an improvement in UI symptoms increased with continued training: after completing roughly 50 games, 71% of users reported in improvement in UI symptoms; this increased to 79% after ∼100 games and to 85% after completing 300 games.

Similarly, the magnitude of UI symptoms decreased significantly with continued training, with progressively greater symptom reduction observed at each timepoint. On average, total symptom score was reduced by about half compared to baseline in women who responded to the symptoms questionnaire at the fourth and final timepoint (T4). Furthermore, the large effect size at medium- and long-term timepoints suggests a strong positive effect of training with the Perifit to reduce UI symptoms, with real relevance for women struggling with UI.

PFMT represents a particularly interesting area for home-based interventions because consensus is widespread that PFMT is effective at treating uncomplicated UI, and should be offered as conservative, first-line treatment to women of all ages.^13,14^ Despite its demonstrated effectiveness, exercise adherence is low.^23^ Additionally, patients report substantial barriers to accessing pelvic floor physical therapy, including cost, time constraints, and issues organizing travel to and from appointments.^28^ Home-based solutions may therefore play a role in expanding access to care.

The improvements observed after training with the Perifit device are comparable to success rates commonly cited in the literature. The most recent update to the Cochrane systematic review of PFMT for the treatment of UI reported that PFMT is effective in reducing UI symptoms in about 74% of women with SUI and about 67% of women with any UI.^13^ A recent systematic review of mobile app-based programs found variable success rates for mobile app-based training, reporting improvement in UI symptoms in 40% to 66% of women at ∼4 months, increasing to 92% of women at 2 years of follow-up.^23^ In the current analysis, the “short-term” and “long-term” improvement timepoints corresponded to training durations averaging 41 days (or about 6 weeks) and 125 days (or about 4 months), respectively. The timing of the reduction in UI symptoms achieved with the Perifit device is therefore in line with previous reports of both clinic- and home-based treatment plans.

Subgroup analysis of responders versus nonresponders to home PFMT with the Perifit revealed that improvement in UI symptoms was unrelated to baseline pelvic floor strength, age, menopausal status, time since childbirth, or the presence of prolapse. However, there was a significant baseline difference in symptom severity between responders and nonresponders. Users reporting more severe symptoms at baseline were more likely to experience symptom reduction. The difference cannot be attributed to training volume, which was standardized to collect comparable data on each user. However, it is possible that this difference may be due to the measurement instrument used to assess UI symptoms. The USP assesses severity of UI symptoms using questions with four possible answers that are sometimes a characterization of frequency; for example, *never; less than once per week; several times per week;* or *several times per day.* Users with higher symptom severity at baseline may be more likely to be able to report a change in response category after PFMT; for example, reducing leaks from *several times per day* to *several times per week.* For users already in the lower symptom categories, such as *less than once per week,* the questionnaire may lack the necessary granularity to detect symptom improvement. An objective assessment of leaks using a 24 h pad test, for example, may have produced a different result.

The effect size of the improvement in UI symptoms after training with the Perifit device was medium (at short term) to large (medium to long term). This is especially relevant because UI symptoms affect between one quarter and one half of all women,^3–6^ and research has documented that most women with UI symptoms do not discuss UI symptoms with their health care professional. Common reasons include shame and embarrassment, the belief that their symptoms are minor or a “normal” consequence of childbirth or aging, and not wanting to waste their doctor's time.^4,17,18^

Importantly, the real-world consequences of UI reach beyond simple pelvic floor dysfunction: women with UI may reduce or stop their participation in preferred physical activities,^7,8^ experience negative impacts on their sexuality and sexual function,^17^ and bear a substantial financial burden associated with physician's visits, the purchase of incontinence and odor-control products, and costs associated with laundry and dry-cleaning.^9,10,29,30^ Additionally, as incontinence is more frequent in women, these costs are also disproportionately borne by women. Home-based, over-the-counter options for structured PFMT address some of the barriers to accessing UI-related health care and are thus an important lever for reducing gender gaps in care.

The real-world data design presents some advantages as well as some substantial limitations. As an observation of real-world use of a commercially available, over-the-counter device, this analysis demonstrates that performing structured PFMT using the Perifit device and its accompanying app may be an effective option for the self-management of UI symptoms in real-world conditions. The percentage of women who perform home training with the Perifit device and experience UI symptom reduction is comparable to what is observed in more controlled environments.

An additional strength was the use of a validated questionnaire to assess UI symptoms. The analysis also included an exceptionally wide age range (18–87 years), and the absence of an effect of age, menopausal status or other baseline characteristics on symptom reduction suggests that home-based PFMT with the Perifit may improve PFMT symptoms in a broad range of women. Finally, the sample size was far larger than most published trials, and apart from setting training volume thresholds for including questionnaire responses in the short-, medium-, and long-term groups, no data were omitted, making this dataset quite representative of the range of users of the Perifit device in the real world.

However, there are also some substantial drawbacks to the real-world data design: first, because the questionnaire is an optional feature within the Perifit smartphone app, not all users of the Perifit device complete the questionnaire at the *a priori* defined timepoints for inclusion in the short-, medium- or long-term analysis. There is certainly a selection bias in the dataset; for example, it is possible that women who felt more positively about their symptom evolution were more likely to continue to train and to complete the questionnaire regularly to document their progress. We attempted to reduce the effect of this bias by proposing the questionnaire as a pop-up prompt to all individuals who opened the app to train and who had reached the target number of games, and by using a questionnaire that quantifies symptoms objectively instead of asking for a subjective rating of improvement. Nonetheless, despite these mitigation measures, we cannot discount a selection bias.

Furthermore, since users of the device had to reach the threshold number of completed games to be included in the short-, medium-, or long-term analyses, users who stopped using the device before reaching 40 games, as well as users who may have continued to train but who did not complete the questionnaire during the designated game windows were not included in any aspect of this analysis. We recognize the limitation of this design. In designing this analysis, we considered binning users either by calendar days since beginning training (elapsed training time), or by the number of games played (training volume). Ultimately, since training volume is key to improving muscle strength and function, the current analysis focused on the number of games completed instead of a specific duration for follow-up. This allowed us to compare individuals who had completed the same training volume and accounted for the fact that in the real world, users will train with the device at different frequencies.

Additionally, this analysis was unable to follow users who did not continue to train with the device. Previous research has suggested that adherence to home-based PFMT is variable, and that patient-related factors including perceived self-efficacy,^31^ having adequate knowledge of the benefits of PFMT, knowing an incontinent woman of the same age, and having a regular cue to exercise influence exercise adherence.^32^ While the app-based environment of the Perifit includes features that align with these factors, the current analysis did not assess the adherence dimension of PFMT. Much like the effectiveness of exercise on cardiovascular fitness would not be assessed by evaluating users who had purchased and worn athletic shoes at least once, the effectiveness of PFMT on UI symptoms depends on users completing a certain amount of training. In this sense, this study was limited to assessing the real-world effectiveness of the Perifit device in users *who actually trained with the device*.

It is also important to note that women who purchased the Perifit device are not necessarily representative of all segments of the population. Women needed to be aware that their pelvic floor symptoms were potentially treatable with PFMT, be able to afford to purchase the device, be able to dedicate 10–15 minutes per training session to performing pelvic floor exercise, and be sufficiently literate to understand the symptoms questionnaire and respond. Further work is needed to better understand how digital devices may be used to reach populations who are less likely to seek treatment for pelvic floor dysfunction or who face other barriers to exercise adherence.

Finally, while PFMT is broadly endorsed as first-line, conservative therapy for UI symptoms, there are certain populations for whom PFMT, with or without a device, is not necessarily recommended or effective. It is often thought that UI is caused by weak pelvic floor muscles, but counterintuitively, hypertonicity of the pelvic floor may also cause UI symptoms. In this case, PFMT may exacerbate symptoms. Moreover, some women with hypertonicity, vaginismus, or vaginal atrophy may find the insertion of a biofeedback device for home training to be painful or impossible. Therefore, while home-based training may remove some barriers to training, it remains important to balance the relative benefits and risks of PFMT in the event of comorbidities or potential contraindications.

In conclusion, this analysis of responses from over 6000 real-world users suggests that home training with the Perifit may be an effective tool to reduce UI symptoms in women of all ages. The percentage of women who reported a quantitative reduction in UI symptoms increased from 71% after completing 50 games (on average, 6 weeks of training), to 85% after completing 300 games (on average, 4 months of training). The effect size of symptom reduction was medium to large, suggesting a relevant impact on UI symptoms in women who adhered to home training. Further research is needed to assess the effectiveness of home-based biofeedback devices on factors such as exercise adherence, motivation, and barriers to exercise. Given the quality of life, economic, and social burdens of living with UI symptoms, home-based PFMT with the Perifit may be a promising tool to allow women to self-manage UI.

## Authors' Contribution

E.T.P.: conceptualization, methodology, formal analysis, writing—original draft, review & editing, data curation, and visualization. L.A.: conceptualization, methodology, software, formal analysis, writing—original draft, and data curation.

## Abbreviations Used

MUI: mixed urinary incontinence
PFMT: pelvic floor muscle training
SUI: stress urinary incontinence
UI: urinary incontinence
USP: urinary symptom profile
UUI: urge urinary incontinence

## References

[1] National Guideline Alliance. Risk factors for pelvic floor dysfunction. Natl Inst Heal Care Excell 2021;PMID:35442598. 35442598

[2] Dumoulin C, Pazzoto Cacciari L, Mercier J. Keeping the pelvic floor healthy. Climacteric 2019;22(3):257–262; doi: 10.1080/13697137.2018.1552934 30653374

[3] Lee UJ, Feinstein L, Ward JB, et al. Prevalence of Urinary Incontinence among a Nationally Representative Sample of Women, 2005-2016: Findings from the Urologic Diseases in America Project. J Urol 2021;205(6):1718–1724; doi: 10.1097/JU.0000000000001634 33605795

[4] Hunskaar S, Lose G, Sykes D, et al. The prevalence of urinary incontinence in women in four European countries. BJU Int 2004;93(3):324–330; doi: 10.1111/j.1464-410x.2003.04609.x 14764130

[5] Schreiber Pedersen L, Lose G, Høybye MT, et al. Prevalence of urinary incontinence among women and analysis of potential risk factors in Germany and Denmark. Acta Obstet Gynecol Scand 2017;96(8):939–948; doi: 10.1111/aogs.13149 28401541

[6] MacLennan AH, Taylor AW, Wilson DH, et al. The prevalence of pelvic floor disorders and their relationship to gender, age, parity and mode of delivery. BJOG 2000;107(12):1460–1470; doi: 10.1111/j.1471-0528.2000.tb11669.x 11192101

[7] Dakic JG, Cook J, Hay-Smith J, et al. Pelvic floor disorders stop women exercising: A survey of 4556 symptomatic women. J Sci Med Sport 2021;24(12):1211–1217; doi: 10.1016/j.jsams.2021.06.003 34244084

[8] Salvatore S, Serati M, Laterza R, et al. The impact of urinary stress incontinence in young and middle-age women practising recreational sports activity: An epidemiological study. Br J Sports Med 2009;43(14):1115–1118; doi: 10.1136/bjsm.2008.049072 18819959

[9] Subak LL, Brown JS, Kraus SR, et al. The “costs” of urinary incontinence for women. Obstet Gynecol 2006;107(4):908–916; doi: 10.1097/01.AOG.0000206213.48334.09 16582131 PMC1557394

[10] Papanicolaou S, Pons ME, Hampel C, et al. Medical resource utilisation and cost of care for women seeking treatment for urinary incontinence in an outpatient setting. Examples from three countries participating in the PURE study. Maturitas 2005;52 (Suppl 2):S35-47; doi: 10.1016/j.maturitas.2005.09.004 16297577

[11] Salonia A, Zanni G, Nappi RE, et al. Sexual dysfunction is common in women with lower urinary tract symptoms and urinary incontinence: Results of a cross-sectional study. Eur Urol 2004;45(5):642–648; discussion 648; doi: 10.1016/j.eururo.2003.11.023 15082208

[12] Duralde ER, Rowen TS. Urinary incontinence and associated female sexual dysfunction. Sex Med Rev 2017;5(4):470–485; doi: 10.1016/j.sxmr.2017.07.001 28827036

[13] Dumoulin C, Cacciari LP, Hay-Smith EJC. Pelvic floor muscle training versus no treatment, or inactive control treatments, for urinary incontinence in women. Cochrane Database Syst Rev 2018;10(10):CD005654; doi: 10.1002/14651858.CD005654.pub4 30288727 PMC6516955

[14] Abrams P, Andersson K-E, Apostolidis A, et al. 6th International Consultation on Incontinence. Recommendations of the International Scientific Committee: Evaluation and treatment of urinary incontinence, pelvic organ prolapse and faecal incontinence. Neurourol Urodyn 2018;37(7):2271–2272; doi: 10.1002/nau.23551 30106223

[15] Bump RC, Hurt WG, Fantl JA, et al. Assessment of Kegel pelvic muscle exercise performance after brief verbal instruction. Am J Obstet Gynecol 1991;165(2):322–329; doi: 10.1016/0002-9378(91)90085-6 1872333

[16] Barton A, Serrao C, Thompson J, et al. Transabdominal ultrasound to assess pelvic floor muscle performance during abdominal curl in exercising women. Int Urogynecol J 2015;26(12):1789–1795; doi: 10.1007/s00192-015-2791-9 26215905

[17] Mendes A, Hoga L, Gonçalves B, et al. Adult women's experiences of urinary incontinence: A systematic review of qualitative evidence. JBI Database Syst Rev Implement reports 2017;15(5):1350–1408; doi: 10.11124/JBISRIR-2017-003389 28498174

[18] Institute for Healthcare Policy and Innovation. National Poll on Healthy Aging. n.d. Available from: https://www.healthyagingpoll.org/reports-more/report/urinary-incontinence-inevitable-part-aging [Last accessed: July 27, 2023].

[19] Wang X, Xu X, Luo J, et al. Effect of app-based audio guidance pelvic floor muscle training on treatment of stress urinary incontinence in primiparas: A randomized controlled trial. Int J Nurs Stud 2020;104:103527; doi: 10.1016/j.ijnurstu.2020.103527 32058140

[20] Löjdahl E, Lindam A, Asklund I. App-based pelvic floor muscle training in pregnant and postnatal women: A prospective cohort study exploring factors associated with prevention and improvement of urinary incontinence. Heal Sci Rep 2022;5(5):e781; doi: 10.1002/hsr2.781 PMC9387900 36000079

[21] Loohuis AMM, Wessels NJ, Dekker JH, et al. App-based treatment in primary care for urinary incontinence: A pragmatic, randomized controlled trial. Ann Fam Med 2021;19(2):102–109; doi: 10.1370/afm.2585 33685871 PMC7939722

[22] Wadensten T, Nyström E, Franzén K, et al. A mobile App for self-management of urgency and mixed urinary incontinence in women: Randomized controlled trial. J Med Internet Res 2021;23(4):e19439; doi: 10.2196/19439 33818395 PMC8056293

[23] Hou Y, Feng S, Tong B, et al. Effect of pelvic floor muscle training using mobile health applications for stress urinary incontinence in women: A systematic review. BMC Womens Health 2022;22(1):400; doi: 10.1186/s12905-022-01985-7 36192744 PMC9531466

[24] Jochum F, Garbin O, Godet J, et al. Prospective evaluation of the connected biofeedback EMY Kegel trainer in the management of stress urinary incontinence. J Gynecol Obstet Hum Reprod 2022;51(2):102280; doi: 10.1016/j.jogoh.2021.102280 34861424

[25] Louin E. Mesure de Pression de Capteurs de La Sonde Périnéale Connectée Perifit Lors d'une Contraction Des Muscles Du Plancher Pelvien et Lors d'une Manoeuvre de Valsalva. Ecole Nationale de Kinésithérapie et Réeducation; 2021. Available from: https://kinedoc.org/work/kinedoc/196fb820-7746-4bc6-807e-80f1f7bb4ac8.pdf [Last accessed: August 3, 2023].

[26] US Department of Health and Human Services. Guidance Regarding Methods for De-Identification of Protected Health Information in Accordance with the Health Insurance Portability and Accountability Act (HIPAA) Privacy Rule. n.d. Available from: https://www.hhs.gov/hipaa/for-professionals/privacy/special-topics/de-identification/index.html [Last accessed: July 27, 2023].

[27] Haab F, Richard F, Amarenco G, et al. Comprehensive evaluation of bladder and urethral dysfunction symptoms: Development and psychometric validation of the Urinary Symptom Profile (USP) questionnaire. Urology 2008;71(4):646–656; doi: 10.1016/j.urology.2007.11.100 18313122

[28] Zoorob D, Higgins M, Swan K, et al. Barriers to pelvic floor physical therapy regarding treatment of high-tone pelvic floor dysfunction. Female Pelvic Med Reconstr Surg 2017;23(6):444–448; doi: 10.1097/SPV.0000000000000401 28145917

[29] Bohorquez J, McKinney J, Keyser L, et al. Development of a wireless accelerometer-based Intravaginal device to detect pelvic floor motion for evaluation of pelvic floor dysfunction. Biomed Microdevices 2020;22(2):26; doi: 10.1007/s10544-020-00479-3 32185505 PMC7078143

[30] Hu T-W, Wagner TH, Bentkover JD, et al. Costs of urinary incontinence and overactive bladder in the United States: A comparative study. Urology 2004;63(3):461–465; doi: 10.1016/j.urology.2003.10.037 15028438

[31] Sacomori C, Berghmans B, de Bie R, et al. Predictors for adherence to a home-based pelvic floor muscle exercise program for treating female urinary incontinence in Brazil. Physiother Theory Pract 2020;36(1):186–195; doi: 10.1080/09593985.2018.1482583 29863450

[32] Cooper H, Carus C. Factors affecting women's adherence to pelvic floor muscle exercises in a first pregnancy: A qualitative interview study. J Pelvic Obstet Gynaecol Physiother 2015;117:29–34.

